# Evidence for a novel functional role of cannabinoid CB_2_ receptors in the thalamus of neuropathic rats

**DOI:** 10.1111/j.1460-9568.2008.06162.x

**Published:** 2008-04-01

**Authors:** M D Jhaveri, S J R Elmes, D Richardson, D A Barrett, D A Kendall, R Mason, V Chapman

**Affiliations:** 1 School of Biomedical Sciences, Medical School, University of Nottingham Nottingham NG7 2UH, UK; 2 School of Pharmacy, University of Nottingham Nottingham NG7 2UH, UK; 3 BioScience (2213a), AstraZeneca R & D Montréal 7171 Frédérick-Banting, Ville Saint-Laurent (Montréal), Québec Canada H4S 1Z9; 4 Pfizer Limited Ramsgate Road, Sandwich, Kent CT13 9NJ, UK

**Keywords:** electrophysiology, JWH-133, neuropathic pain, spinal nerve ligation, VPL

## Abstract

Cannabinoid CB_1_ receptors have analgesic effects in models of neuropathic pain, but can also produce psychoactive side-effects. A supraspinal location of CB_2_ receptors has recently been described. CB_2_ agonists are also antinociceptive, although the functional role of supraspinal CB_2_ receptors in the control of nociception is unknown. Herein, we provide evidence that CB_2_ receptors in the thalamus play a functional role in the modulation of responses of neurons in the ventral posterior nucleus (VPL) of the thalamus in neuropathic, but not sham-operated, rats. Spontaneous and mechanically evoked activity of VPL neurons was recorded with a multichannel electrode array in anaesthetized spinal nerve-ligated (SNL) rats and compared to sham-operated rats. Intra-VPL administration of the CB_2_ agonist JWH-133 (30 ng in 500 nL) significantly reduced spontaneous (*P* < 0.05), non-noxious (*P* < 0.001) and noxious (*P* < 0.01) mechanically evoked responses of VPL neurons in SNL rats, but not in sham-operated rats. Inhibitory effects of JWH-133 on spontaneous (*P* < 0.01) and noxious-evoked (*P* < 0.001) responses of neurons were blocked by the CB_2_ antagonist SR144528. Local administration of SR144528 alone did not alter spontaneous or evoked responses of VPL neurons, but increased burst activity of VPL neurons in SNL rats. There were, however, no differences in levels of the endocannabinoids anandamide and 2AG in the thalamus of SNL and sham-operated rats. These data suggest that supraspinal CB_2_ receptors in the thalamus may contribute to the modulation of neuropathic pain responses.

## Introduction

Neuropathic pain states have complex aetiologies and are difficult to treat with analgesics. Studies in animal models of neuropathic pain suggest that cannabinoid receptors are a potential therapeutic target. Both systemic and local administration of cannabinoid 1 receptor (CB_1_) agonists attenuate pain behaviour and evoked neuronal responses in neuropathic rats (Fox *et al*., 2001; Costa *et al*., 2004; Scott *et al*., 2004; Sagar *et al*., 2005; Liu & Walker, 2006). CB_1_ receptors are, however, densely expressed throughout the brain and their activation is associated with psychoactive side-effects.

Systemic administration of selective cannabinoid 2 receptor (CB_2_) agonists is also analgesic in models of inflammatory (Hanus *et al*., 1999; Clayton *et al*., 2002; Nackley *et al*., 2003; Valenzano *et al*., 2005; Whiteside *et al*., 2005; Beltramo *et al*., 2006; Sanson *et al*., 2006) and neuropathic (Ibrahim *et al*., 2003; Scott *et al*., 2004; Valenzano *et al*., 2005; Whiteside *et al*., 2005; Beltramo *et al*., 2006) pain, in the absence of central nervous system (CNS)-mediated side-effects. Until recently, dogma held that CB_2_ receptors were not present in the spinal cord or brain (Howlett, 1995; Pertwee, 1997) and effects of CB_2_ agonists were assumed to arise as a result of activation of peripheral CB_2_ receptors. This dichotomy between CB_2_ and CB_1_ receptor expression in the CNS has, however, been dispelled by reports of CB_2_ receptor expression in the brainstem (Van Sickle *et al*., 2005) and higher brain centres, including the thalamus (Gong *et al*., 2006) and cerebellum (Ashton *et al*., 2006) in naïve rats, although their physiological roles are unclear. Thus, although it has been assumed that the effects of CB_2_ agonists arise as a result of activation of receptors on peripheral immune cells and, under some pathological conditions, on microglia (Carlisle *et al*., 2002; Benito *et al*., 2003; Facchinetti *et al*., 2003; Klegeris *et al*., 2003; Walter *et al*., 2003; Carrier *et al*., 2004; Nunez *et al*., 2004; Cabral & Marciano-Cabral, 2005), the presence of cells expressing these receptors in the brain suggests that they might mediate some of the analgesic effects of systemically administered CB_2_ agonists. Previously, we have demonstrated a novel functional role of spinal CB_2_ receptors in modulating nociceptive processing in neuropathic, but not sham-operated, rats (Sagar *et al*., 2005), supporting their presence in the spinal cord of neuropathic rats (Zhang *et al*., 2003; Wotherspoon *et al*., 2005; Beltramo *et al*., 2006).

On the basis of the description of supraspinal localization of CB_2_ receptors and the well-accepted role of structures such as the thalamus (Guilbaud *et al*., 1990; Bordi & Quartaroli, 2000) contributing to neuropathic states, we hypothesized that supraspinal CB_2_ receptors in the thalamus might contribute to inhibitory effects of these ligands in neuropathic states; this would have important implications for the future clinical use of this class of compounds. Here, we report that intrathalamic administration of a selective CB_2_ agonist, JWH-133, attenuates spontaneous activity and evoked responses of neurons of the ventral posterior nucleus of the thalamus (VPL) in neuropathic, but not sham-operated, rats. These data provide evidence for a functional role of supraspinal CB_2_ receptors in the modulation of neuronal responses in neuropathic rats.

## Materials and methods

### Spinal nerve-ligated (SNL) rats

Experiments were carried out on male Sprague–Dawley rats (*n* = 45; Charles River, UK) weighing 140–150 g at the beginning of the study. Rats were housed five per cage under a 12 : 12 h day : night cycle for 1 week prior to surgery. Rats were divided into two groups: SNL group (ligation of the L5–L6 spinal nerves; *n* = 31) and sham group (*n* = 14). All described procedures were approved by the Home Office, UK and follow the guidelines of the International Association for the Study of Pain.

The procedure of ligation of the spinal nerves was performed as previously described by Kim & Chung (1992). Briefly, under isoflurane anaesthesia (1.5–2% in 66% N_2_O, 34% O_2_) the rat was placed in a prone position and the left paraspinal muscles were separated from the spinous processes at the L4–S2 level. Part of the L6 transverse process was carefully removed and the L4–L6 spinal nerves were identified. The L5–L6 spinal nerves were isolated and tightly ligated, distal to the dorsal root ganglion and proximal to the formation of the sciatic nerve, with 6–0 silk thread. Following complete haemostasis the wound was sutured. The total period of anaesthesia did not exceed 30 min. The procedure for the sham operation for spinal nerve ligation was identical to that of the SNL group, except that the spinal nerves were exposed but not ligated.

After surgery, the rats were maintained under the same conditions as during the preoperative period. The posture and behaviour of the rats were carefully monitored following recovery from the anaesthesia and then on the first postoperative day. From postoperative day 2 onwards, behavioural tests assessing sensitivity to mechanical stimuli were performed for up to 14 days. The weight gain and general behaviour of the rats was monitored throughout the postoperative period.

### Behavioural testing of allodynia

Based on our previous experience and on aberrant pain responses associated with clinical neuropathic pain, mechanical allodynia was considered the most appropriate behavioural measure for these studies. Rats were placed in transparent plastic cubicles on a mesh-floored table and a period of acclimatization was allowed prior to testing. The mechanical sensitivity of the ipsilateral and contralateral hindpaws of SNL or sham-operated rats was assessed by measuring the frequency of withdrawal of the hindpaw to mechanical punctate stimuli (calibrated von Frey filaments; calibration codes 4.08, 4.56 and 5.07, corresponding to bending forces of 1, 4 and 10 g, respectively). Stimuli were applied to the plantar surface of the hindpaw and each trial consisted of the application of a single von Frey hair 10 times in descending force order, each for a period of 2–3 s. Testing with consecutive von Frey hairs was separated by a period of at least 5 min. The occurrence of hindpaw withdrawal for each trial was expressed as a percentage response frequency (10 × total number of hindpaw withdrawals to ten applications).

### In vivo *electrophysiology*

Methods used were similar to those previously described by Abdul Aziz *et al*. (2005). Two weeks following sham surgery or spinal nerve ligation, rats were anaesthetized for electrophysiological studies. Anaesthesia was induced with 3% isoflurane in a 50% N_2_O : 50% O_2_ mixture. The level of isoflurane was reduced progressively and maintained at 1.7–2.2% throughout surgery to ensure a state of complete areflexia. Rats were subsequently mounted in a modified stereotaxic frame. Core temperature was monitored and maintained between 37 and 38 °C using a homeothermic heating pad (Harvard Instruments, UK). A scalp incision was made, and a 5-mm-diameter craniotomy was performed. The cortex above the thalamus was exposed, the dura mater was excised and exposed tissue was kept moist with saline solution (0.9% NaCl).

### Recording procedure

An eight-microwire electrode array (2 × 4 array, 0.2 mm wide × 0.7 mm long; Teflon-coated stainless steel, 50 µm diameter per wire; NB Laboratories, TX, USA) with impedances of 100–300 kΩ measured at 1 kHz (Robinson & Mishkin, 1968), complete with microinjector (∼ 150-µm-diameter stainless steel cannula, < 500 µm above the recording electrode wire tips) to allow for local drug administration through pressure ejection at the electrode recording tip (Krupa *et al*., 1999), was used to record spike activity from a number of single VPL neurons. Electrodes were placed in the VPL (3.2 mm posterior, 3.0–3.2 mm lateral and 5.0–7.0 mm ventral from bregma) of the thalamus on the side contralateral to spinal nerve ligation, according to the atlas of Paxinos & Watson (1997). The electrode assembly was clamped to a Narishige manipulator which was used to progressively lower the array through the right thalamus. The electrode-injector array was gradually lowered over ∼ 2 h to reach 5.0 mm ventral from bregma to minimize any trauma caused by the insertion of the array. Subsequent lowering was carried out in tandem with mechanical stimulation of the nerve-injured hindpaw to identify mechanically sensitive VPL neurons.

The electrode array was connected via an eight-channel unity-gain headstage to a multichannel preamplifier (gain ×1000, bandpass-filtered 150 Hz−9 kHz; Plexon Inc; http://www.plexoninc.com). Extracellular action potential spikes were then fed to a Plexon Multichannel Acquisition Processor system linked to a host PC, providing simultaneous 40-kHz (25-µs) A/D conversion on each channel at 12-bit resolution and additional programmable amplification and filtering of spikes (final bandwidth 400Hz−5 kHz). Spike discrimination on-line was achieved with pairs of voltage–time windows and principal component analysis (Abeles & Goldstein, 1977); typically, 5–8 electrodes in the array picked up neuronal activity and 1–3 spikes could be confidently discriminated per electrode, giving a range of 8–15 isolated VPL neurons.

### Peripheral cutaneous stimulation

All neurons studied in the right VPL had receptive fields on the plantar region of the left (nerve-injured) hindpaw and responded to innocuous (7 g) and noxious (65 g) mechanical stimuli, indicating that these neurons were wide dynamic range neurons. The spontaneous activity of neurons was recorded throughout the study, 3 min prior to recording of mechanically evoked responses, i.e pre- and 12, 27, 42 and 57 min postdrug injection. All evoked responses were normalized by subtracting the spontaneous activity of the individual neuron. The noxious withdrawal threshold in awake animals is 15 g (Chaplan *et al*., 1994); thus, weights exceeding the 15-g threshold are described as noxious. A calibrated pneumatic probe (SMC miniature actuation cylinder, RS components product no. 264–6119, circular contact area of 1 mm diameter) was used to deliver trains of innocuous (7 g) or noxious (65 g) stimulation to the peripheral receptive field of VPL neurons on the nerve-injured hindpaw. Pressure and timing of the probe was controlled via a pneumatic picopump (WPI model Pv800; USA). Through the clamping of the pneumatic probe in a fixed position a train of calibrated mechanical stimulation was accurately delivered to the same point on the hindpaw receptive field, eradicating any experimenter-induced variation. Peri-stimulus time histograms (PSTHs) of innocuous (7 g) and noxious (65 g) mechanically evoked responses were computed using NeuroExplorer (Nex Technologies, USA). A train of mechanical stimulation consisted of 20 stimuli of 5 s duration over a 5-min time period (15-s window between the initiation of successive stimuli). In order to minimize any desensitization produced by the repeated mechanical stimulus trains, innocuous (7 g) and noxious (65 g) stimulation were alternated so that application of noxious stimuli was separated by at least 30 min.

### Pharmacological studies

Prior to any pharmacological intervention, control basal spontaneous activity and control innocuous and noxious mechanically evoked PSTHs were recorded. The effects of a local VPL administration (via pressure ejection through a microinjector over a 2-min period) of the selective CB_2_ receptor agonist JWH-133 (30 ng in 500 nL, 192 µm) alone, JWH-133 and the selective CB_2_ receptor antagonist SR144528 (respectively, 30 and 48 ng in 500 nL, administered as a single infusion; 192 and 201 µm), SR144528 (48 ng in 500 nL) administered alone and vehicle (0.3% ethanol, 0.3% Tween 80 in artificial cerebrospinal fluid) on spontaneous burst activity and mechanically evoked VPL neuronal responses was investigated. The effects of a local VPL administration of JWH-133 (30 ng in 500 nL, 192 µm) and the selective CB_1_ receptor antagonist AM251 (respectively, 30 and 48 ng in 500 nL, administered as a single infusion; 192 and 172 µm) on mechanically evoked VPL neuronal responses was also investigated in SNL rats. Agonist and antagonist drugs were administered as a single infusion due to the constraints of using a microinjector that was preloaded prior to implantation and recording. Doses of the drugs used in this study were based on previous reports of spinal and supraspinal administration of cannabinoid ligands (Lichtman *et al*., 1996; Martin *et al*., 1999; Sagar *et al*., 2005).

### Measurement of levels of endocannabinoids in the thalamus

Separate groups of SNL (*n* = 4) and sham-operated (*n* = 4) rats were used for the measurement of levels of endocannabinoids using a previously validated method (Richardson *et al*., 2007). In brief, the thalamus was dissected from rats killed by decapitation and stored at −80 °C for two to four weeks prior to spectrometric analysis for measurement of endocannabinoids and related compounds. Further subdivision of the thalamus was not possible due to the requirement for the rapid removal of tissue to prevent any post-mortem effects on levels of endocannabinoids measured and the amount of tissue required for reliable analysis. Endocannabinoids were extracted using a lipid extraction method; tissue was homogenized in an ethyl acetate–hexane mixture with internal standards added in fixed amounts to all samples (0.42 nmol d8-anandamide, 1.5 nmol d8–2-arachidonyl glycerol, 0.2 nmol heptadecanoyl ethanolamide), followed by repeated centrifugation and supernatant collection stages. Solid-phase extraction was subsequently performed to purify samples. Simultaneous measurement of endocannabinoids and related compounds was then performed using liquid chromatography–tandem mass spectrometry. Analysis was carried out on an Agilent 1100 system coupled to a triple quadrupole Quattro Ultima mass spectrometer (Waters, UK) recording in electrospray-positive mode. Analytes were separated chromatographically on a HyPurity Advance C8 column and precolumn (100 × 2.1 mm internal diameter, 3 µm particle size) with a mobile phase flow rate of 0.3 mL/min. A gradient elution was used, with mobile phases consisting of A (water with 1 g/L ammonium acetate and 0.1% formic acid) and B (acetonitrile with 1 g/L ammonium acetate and 0.1% formic acid). Samples were injected from a cooled autosampler maintained at 4 °C. Multiple reaction monitoring of individual compounds, using specific precursor and product mass-to-charge (*m/z*) ratios allowed simultaneous measurement of anandamide (AEA), 2-arachidonyl glycerol (2AG), palmitoyl ethanolamide (PEA) and oleoyl ethanolamide (OEA). The peak area of each analyte was divided by the appropriate internal standard peak area, and this analyte/internal standard ratio was used to achieve quantification by the internal standard method. Individual calibration lines were obtained during each analytical run by applying the method to a suitable range of concentrations of the nondeuterated forms of each analyte. Data are reported only for analytes above the limit of quantification by this method (10 pmol/g except for 2AG, 100 pmol/g).

### Histology

At the end of each experiment, rats were overdose-killed with isoflurane and a current (10 µA) was passed for 10 s through a single electrode in the middle of the array to deposit ferric ions at the recording site. Brains were removed and stored overnight in 4% paraformaldehyde. Tissue blocks were sectioned transversely at 100 µm using a vibrotome (Campden Instruments, UK). Iron deposits at the electrode tips were revealed by the Prussian Blue reaction (Hong *et al*., 2000). Recording sites were identified with reference to the rat brain atlas of Paxinos & Watson (1997).

### Data analysis

For each neuron isolated, the mean spontaneous frequency of firing was recorded over a period of 3 min just prior to mechanical stimulation. To allow for comparisons between the absolute magnitudes of evoked responses of neurons, spontaneous activity (spikes/s) of each neuron was subtracted from mechanically evoked responses of neurons (spikes/s). PSTHs were plotted from trains of alternating innocuous (7 g) and noxious (65 g) mechanical stimuli, plotting total activity (spikes/s) generated by 20 × 5 s stimulations. Effects of the various pharmacological treatments on spontaneous and evoked activity are expressed as a percentage of control responses.

Burst activity (bursts/minute) of neurons was also analysed. A burst was defined using the following criteria in NeuroExplorer software (version 3): maximum interval to start burst, i.e. the first interspike interval within a burst, must be ≤ 6 ms; maximum interval to end burst, 9 ms; minimum interval between bursts, 100 ms; minimum duration of burst, 10 ms; and minimum number of spikes within a burst, two (Fanselow *et al*., 2001; Hains *et al*., 2006).

Data were analysed off-line using Off-Line Sorter (Plexon Inc, USA), NeuroExplorer (version 3.1x, Nex Inc, USA), Matlab (version 7.4; Mathworks, USA), Prism (version 3.03; GraphPad, USA). Statistical analysis was carried out using Mann–Whitney, Kruskall–Wallis and Dunn's *post hoc* test or repeated-measures anova where appropriate. Data are expressed as mean ± SEM; statistical significance was taken as *P* < 0.05.

## Results

### Development of mechanical allodynia in SNL rats

Following SNL surgery, rats exhibited normal grooming behaviour and weight gain similar to sham-operated controls. The development of mechanical allodynia was assessed for up to 14 days after surgery. SNL rats exhibited significant mechanical allodynia. Application of a 10-g mechanical stimulus to the ipsilateral hindpaw of SNL rats evoked a significant increase in paw withdrawal (56 ± 5% paw withdrawal; *P* < 0.001, *n* = 27), compared with sham-operated rats (1 ± 1% paw withdrawal; *n* = 10) on day 10 post-surgery. Similarly, at day 14 post-SNL surgery, application of a 10-g mechanical stimulus to the ipsilateral hindpaw of SNL rats evoked a significant increase in paw withdrawal (48 ± 5% paw withdrawal; *P* < 0.001, *n* = 27), compared with sham-operated rats. In contrast, application of a 10-g mechanical stimulus to the contralateral hindpaw of SNL and sham-operated rats did not evoke a significant paw withdrawal response (*n* = 27 and 10, respectively). SNL rats which were used in the subsequent electrophysiological studies had established mechanical allodynia of the ipsilateral hindpaw, which is in agreement with previous studies of this model (Kim & Chung, 1992; Chapman *et al*., 1998; Jhaveri *et al*., 2006).

### Extracellular electrophysiological recordings from VPL neurons

#### Spontaneous and mechanically evoked neuronal activity in the VPL of sham-operated and SNL rats

**Fig. 1:**
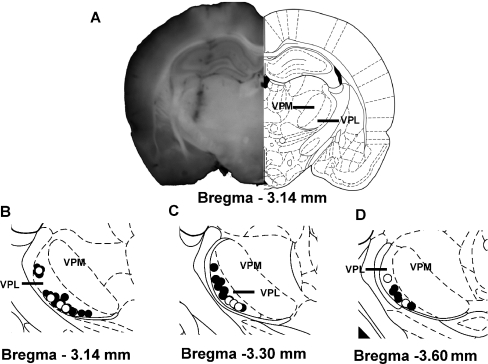
Recording sites of VPL neurons, contralateral to sham surgery or spinal nerve ligation, with mechanical receptive field located on the hindpaw. (A) Representative cannula track diagram and (B–D) histologically identified recording sites at three rostrocaudal coordinates in the VPL of SNL (solid circles) and sham (unfilled, white circles). Each circle represents recording sites in distinct experiments. VPM, ventral posteromedial nucleus; VPL, ventral posterolateral nucleus. Note that there was an overlap in some recording sites and, thus, the number of dots may appear to be fewer than the total number of rats used in this study.

The activity of neurons in the VPL contralateral to SNL (*n* = 414 neurons, 27 rats) or sham surgery (*n* = 99 neurons, 10 rats) was recorded; the location of these neurons was histologically identified as being in the VPL (Fig. 1).

**Fig. 2:**
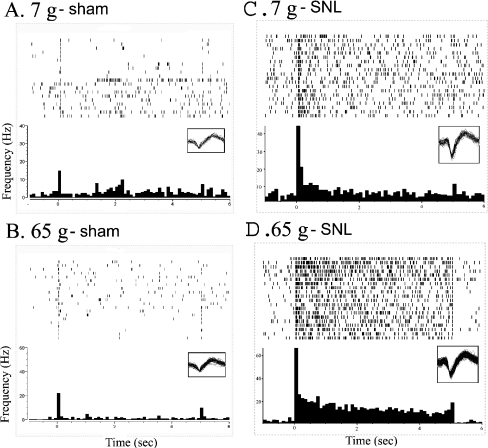
Spontaneous and innocuous (7 g) or noxious (65 g) mechanically evoked VPL neuronal activity in (A and B) sham-operated and (C and D) SNL rats prior to pharmacological intervention, presented as a raster plot and PSTH from the same neuron. An innocuous (7 g) or noxious (65 g) mechanical stimulus was applied to the hindpaw receptive field for 5 s, repeated 20 times at 15-s intervals over a 5-min period, and the activity of VPL neurons on the side contralateral to SNL or sham surgery was recorded. Mechanical stimulus trains were applied every 15 min and alternated between application of an innocuous (7 g) and a noxious (65 g) mechanical stimulus. Raster lines represent responses to 20 individual mechanical stimuli and the PSTH is an average response to those stimuli from a single neuron.

**Fig. 3:**
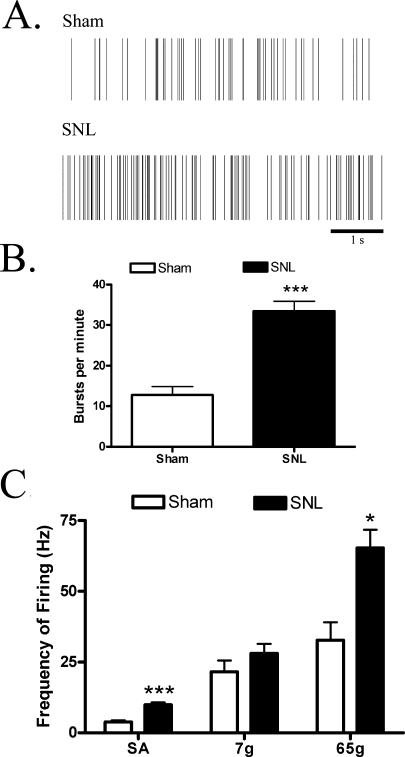
VPL thalamic neurons in SNL rats exhibited more spontaneous activity (SA) and a larger noxious mechanically evoked response than did sham rats. SNL rats also displayed more spontaneous burst activity. (A) Representative raster plot of a neuron from a sham-operated and SNL rat during a burst episode; (B) mean burst activity; and (C) mean spontaneous and evoked responses of VPL neurons. An innocuous (7 g) or a noxious (65 g) mechanical stimulus was applied to the hindpaw receptive field for 5 s, repeated 20 times at 15-s intervals over a 5-min period, and the activity of VPL neurons on the side contralateral to SNL or sham surgery was recorded. Data were analysed using Mann–Whitney test: *P < 0.05, ***P < 0.001 vs. sham (sham, *n* = 47–99 neurons in 10 rats; SNL, *n* = 303–414 neurons in 27 rats).

The spontaneous firing of VPL neurons was of a significantly higher frequency in SNL rats than in sham-operated rats (9.9 ± 0.8 and 3.8 ± 0.5 Hz, respectively; *P* < 0.001; Figs 2 and 3). In SNL rats, 88% of neurons showed burst activity compared to 62% of neurons in sham-operated rats. Furthermore, there was a significantly greater incidence of burst firing of VPL neurons in SNL rats than in sham-operated rats (33.4 ± 2.5 and 12.8 ± 2.1 bursts/min, respectively; *P* < 0.001; Fig. 3). Seventy-four per cent (73/99 neurons) of the spontaneously active VPL neurons recorded on the side contralateral to sham surgery responded to mechanical stimulation of the hindpaw. Similarly, 82% (341/414 neurons) of spontaneously active neurons recorded in the contralateral VPL of SNL rats responded to mechanical stimulation of the nerve-injured hindpaw (Figs 2 and 3). All of the recorded VPL neurons responded to mechanical stimulation of the receptive field on the hindpaw and exhibited a differential magnitude of firing to innocuous (7 g) vs. noxious (65 g) mechanical stimulation (Figs 2 and 3). Mechanically evoked responses of VPL neurons were significantly greater than basal spontaneous activity (*P* < 0.001 for both 7 g and 65 g; Fig. 3). Comparison of evoked responses of VPL neurons revealed that noxious (65 g) evoked responses of VPL neurons were significantly greater in SNL rats than in sham-operated rats (*P* < 0.05; Fig. 3C). Basal spontaneous activity was subtracted from mechanically evoked neuronal activity in order to obtain an absolute value for mechanically evoked neuronal responses. Thus, the marked increase in spontaneous neuronal activity in SNL rats did not contribute to the increase in frequency of firing to the noxious mechanical stimulation in SNL rats.

#### Effects of the selective CB_2_ receptor agonist JWH-133 on responses of VPL neurons in sham-operated and SNL rats

**Fig. 4:**
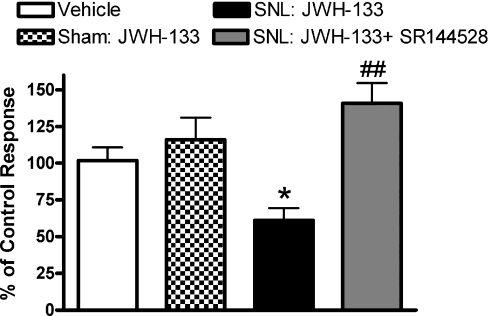
Intrathalamic administration of the CB_2_ receptor agonist JWH-133 inhibited spontaneous activity of VPL neurons in SNL rats. JWH-133 (30 ng in 500 nL, intra-VPL) was administered alone or in combination (single infusion) with the CB_2_ receptor antagonist SR144528 (48 ng). Data were analysed using Kruskall–Wallis test followed by Dunn's *post hoc* test. *P < 0.05 vs. pooled vehicle, ^##^*P* < 0.05 vs. SNL: JWH-133 (*n* = 36–75 neurons in five or six rats per data set). Vehicle treatment data from sham and SNL rats were not statistically different and were pooled for clarity of presentation.

Intrathalamic injection of vehicle produced similar, nonsignificant, effects on neuronal activity in the VPL of sham-operated (*n* = 35 neurons from five rats) and SNL (*n* = 80 neurons from six rats) rats. Effects of local intrathalamic administration of the selective CB_2_ receptor agonist JWH-133 on spontaneous and burst activity of VPL neurons were studied in SNL and sham-operated rats. All data were statistically compared to the appropriate vehicle-treated SNL or sham-operated group. If significance was reached, for clarity of presentation vehicle data for SNL and sham-operated groups were pooled and further statistical analysis compared the effects of drug treatment to the pooled vehicle group. Thirty minutes after administration of JWH-133 (30 ng in 500 nL vehicle) into the thalamus, spontaneous activity of VPL neurons was significantly (*P* < 0.05) inhibited in SNL rats, but not in sham-operated rats, compared to pooled-vehicle controls (Fig. 4). The effects of JWH-133 on the spontaneous activity of VPL neurons were not significant at other timepoints studied. To test the contribution of CB_2_ receptors to the effects of JWH-133, the ability of the CB_2_ receptor antagonist SR144528 to block the effects of JWH-133 was studied in SNL rats. SR144528 (48 ng in 500 nL) significantly blocked the inhibitory effects of JWH-133 on spontaneous activity of VPL neurons in SNL rats (Fig. 4). Intrathalamic administration of SR144528 (48 ng in 500 nL) alone did not significantly alter spontaneous activity of VPL neurons in SNL rats (data not shown).

The effects of JWH-133 on the burst activity of VPL neurons were also studied in sham-operated and SNL rats. In SNL rats, intrathalamic injection of JWH-133 did not significantly alter burst activity of VPL neurons, compared to pooled-vehicle (25-min time-point: vehicle, 69 ± 19% of control; JWH133, 88 ± 30% of control). In addition, JWH133 did not inhibit burst activity of VPL neurons in sham-operated rats (data not shown). Given the lack of effect of JWH133 on burst activity, data from pharmacological studies of coadministered CB_2_ agonist and antagonist were not analysed. We did, however, investigate whether blocking the CB_2_ receptors alone influenced burst activity. In this case, intrathalamic administration of SR144528 (48 ng in 500 nL) significantly (*P* < 0.05) increased burst activity in SNL rats (25-min time-point: 286 ± 137% of control) compared to vehicle (25-min time-point: 69 ± 19% of control) for up to 55 min after drug administration.

**Fig. 5:**
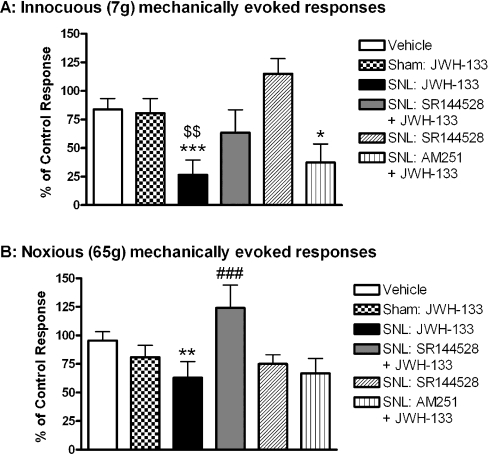
Intrathalamic administration of the CB_2_ receptor agonist JWH-133 inhibited (A) innocuous and (B) noxious mechanically evoked responses of VPL thalamic neurons in SNL rats but not sham-operated rats. JWH-133 (30 ng in 500 nL) was administered intra-VPL, alone or in combination with the CB_2_ receptor antagonist SR144528 (48 ng) or the CB_1_ receptor antagonist AM251 (48 ng) in sham-operated and SNL rats. Innocuous (7 g) and noxious (65 g) mechanically evoked responses were recorded at 15 and 30 min after drug administration, respectively. Data were analysed using the Kruskall–Wallis test followed by Dunn's *post hoc* test. *P < 0.05, **P < 0.01, ***P < 0.001 vs. pooled vehicle; ^###^*P* < 0.001 vs. SNL: JWH-133; ^$$^*P* < 0.01 vs. Sham: JWH-133 (*n* = 46–115 neurons in five or six rats per data set). Vehicle treatment data from sham and SNL rats were not statistically different and were pooled for clarity of presentation.

The ability of the CB_2_ receptor agonist to modulate evoked responses of VPL neurons was also studied. Intra-VPL administration of JWH-133 (30 ng in 500 nL) significantly inhibited innocuous (7 g; *P* < 0.001) and noxious (65 g; *P* < 0.01) mechanically evoked responses of VPL neurons in SNL, but not in sham-operated, rats (Fig. 5). The effects of JWH-133 on 7-g-evoked responses in SNL rats were significantly (*P* < 0.01) different from the effects of JWH-133 in sham-operated rats (Fig. 5A). The CB_2_ receptor antagonist SR144528 (48 ng in 500 nL) significantly (*P* < 0.001) blocked the inhibitory effects of JWH-133 on noxious- but not innocuous-evoked responses of VPL neurons in SNL rats (Fig. 5A and B). Inhibitory effects of JWH-133 on innocuous-evoked responses of VPL neurons were not altered by the CB_1_ receptor antagonist AM251 (48 ng in 500 nL; Fig. 5A). These data provide further support for the role of the CB_2_ receptors in mediating the effects of JWH-133 on evoked responses of VPL neurons.

### Effects of neuropathic pain on endocannabinoids in the VPL

The second part of this study investigated the effects of nerve injury on levels of endocannabinoids in the thalamus of neuropathic rats. Levels of AEA, 2AG, PEA and OEA in the thalamus of SNL and sham-operated rats were quantified 14 days after surgical intervention. We did not observe any significant differences in the levels of these compounds between the thalamus of SNL and sham-operated rats (Table 1). To investigate whether the endocannabinoids can tonically modulate responses of VPL neurons, we also determined the effects of local intrathalamic administration of the selective CB_2_ receptor antagonist SR144528 (48 ng in 500 nL) alone on evoked responses of neurons. Overall, SR144528 did not significantly alter evoked responses of VPL neurons in sham-operated or SNL rats (Fig. 5).

**Table 1:** Levels of endocannabinoids and related compounds in the thalamus of SNL and sham-operated rats

	AEA (pmol/g)	2AG (nmol/g)	OEA (nmol/g)	PEA (nmol/g)
Sham	13.86 ± 1.7	13.38 ± 1.1	0.23 ± 0.02	1.01 ± 0.03
SNL	14.01 ± 2.5	14.38 ± 0.9	0.23 ± 0.05	1.06 ± 0.2

Data are expressed as mean ± SEM pmol/g or nmol/g wet tissue; *n* = 4 each (sham and SNL).

## Discussion

The major finding of the present study was that intra-VPL administration of the selective CB_2_ receptor antagonist JWH-133 attenuated spontaneous and evoked responses of VPL neurons in neuropathic rats but not in sham-operated rats. JWH-133 attenuated both innocuous and noxious mechanically evoked responses of VPL neurons. Inhibitory effects of JWH-133 on spontaneous and noxious-evoked responses of VPL neurons were blocked by the selective CB_2_ receptor antagonist SR144528 but not by the CB_1_ receptor antagonist AM251. This is the first report of a functional effect of supraspinal CB_2_ receptors on neuronal activity in a model of neuropathic pain.

In the present study, VPL neurons exhibited significantly higher spontaneous and burst activity in neuropathic rats than in sham-operated rats. All of the VPL neurons examined in this study responded to innocuous and noxious mechanical stimulation of the peripheral receptive field, indicating that these neurons were wide dynamic range. Noxious mechanically evoked responses of VPL neurons of neuropathic rats were significantly larger than evoked responses in sham-operated rats, corroborating earlier studies in neuropathic rats (Guilbaud *et al*., 1990) and a model of spinal cord injury (Hains *et al*., 2006).

In contrast to the well described spinal and supraspinal distribution of the CB_1_ receptor, until recently the CB_2_ receptor has been predominantly associated with a peripheral distribution. Recent studies in naïve rats reported expression of CB_2_ receptors in the cerebellum, hippocampus, cortex, thalamus, amygdala, substantia nigra, periaqueductal grey matter and inferior colliculi, albeit at levels much lower than those of CB_1_ receptors (Van Sickle *et al*., 2005; Ashton *et al*., 2006; Gong *et al*., 2006). The expression of CB_2_ receptors at sites which modulate pain processing, such as the periaqueductal grey matter and thalamus (Gong *et al*., 2006), suggests that this receptor may modulate nociceptive neurotransmission, either directly or indirectly. In the present study, local administration of a CB_2_ receptor agonist did not modulate spontaneous or evoked responses of VPL neurons in sham-operated rats, suggesting little functional control of neuronal responses at this level under nonpathological conditions. Previously, intralateral posterior thalamic microinjection of the mixed CB_1_ and CB_2_ receptor agonist WIN55212 attenuated nociceptive behavioural processing in naïve rats (Martin *et al*., 1999), although the receptor mediating this effect was not further investigated. In other brain regions, such as the amygdala, the antinociceptive effects of intrabasolateral amygdala injection of WIN55212 in formalin and tail-flick tests were abolished by CB_1_ receptor antagonism but not CB_2_ receptor antagonism in naïve rats (Hasanein *et al*., 2007). Further support for a lack of functional role of supraspinal CB_2_ receptors was provided by the report that intracerebroventricular injection of JWH-133 did not alter formalin-evoked nociceptive responses (Jafari *et al*., 2007). A recent functional magnetic resonance imaging study reported that the selective CB_2_ agonist AM1241 did not alter brain activation in rats, whereas a CB_1_/CB_2_ receptor agonist produced dose-related changes in brain activity (Chin *et al*., 2008). Overall, data from a variety of experimental approaches suggest that there is little contribution of supraspinal CB_2_ receptors to the control of nociceptive processing in naïve or inflamed rats.

In contrast to the lack of effects of the CB_2_ receptor agonist JWH-133 on responses in sham-operated rats, local administration of JWH-133 into the thalamus significantly attenuated the spontaneous activity, but not burst activity, of VPL neurons in neuropathic rats. The relevance of this differential effect of JWH-133 on spontaneous vs. burst activity is presently unclear. Furthermore, the CB_2_ receptor agonist also attenuated both innocuous- and noxious-evoked responses of VPL neurons in neuropathic rats. It is important to note that these inhibitory effects do not merely reflect the attenuation of spontaneous activity, which was subtracted from evoked responses prior to assessment of drug effects. The ability of the CB_2_ receptor agonist JWH-133 to attenuate the facilitated spontaneous activity of VPL neurons, as well as inhibiting both innocuous- and noxious-evoked responses of VPL neurons, in neuropathic rats, suggests that supraspinal CB_2_ receptors may contribute to the inhibitory effects of systemically administered CB_2_ receptor agonists on mechanical allodynia in neuropathic rats (Ibrahim *et al*., 2003; Scott *et al*., 2004; Valenzano *et al*., 2005; Whiteside *et al*., 2005; Beltramo *et al*., 2006). Caution is required in relation to the anatomical interpretation of these data. Although the cannulae were positioned in the VPL, further objective measurements of the spread of the JWH-133 into adjacent brain areas would be required to discount additional local sites of action. Absolute confirmation of these data would require supraspinal electrophysiological studies in neuropathic CB_2_ gene-deleted mice, a combined approach which, to our knowledge, has yet to be applied.

Overall, our data suggest that, following peripheral nerve injury, there are changes in the functional role of CB_2_ receptors in the thalamus which can then be targeted by JWH-133 to modulate responses of VPL neurons. The novel functional role of CB_2_ receptors in the VPL of neuropathic rats may arise as a result of increased receptor expression or increased coupling of pre-existing receptors to their signal transduction systems. Indeed, CB_2_ receptor protein was shown to be increased in the afferent terminals of sensory nerves in the superficial laminae of the dorsal horn of the spinal cord after peripheral nerve damage Wotherspoon *et al*. (2005). CB_2_ receptor expression is also induced in the brains of rats subjected to hypoxia–ischemia, although in this situation the receptors appear to be located on non-neuronal cells including resident microglia or invading monocytes (Ashton *et al*., 2007). As there is no evidence for an increased population of neuroimmune cells in the thalamus of neuropathic rats, the most parsimonious explanation of the present data is an increased expression of CB_2_ receptors on thalamic neurons, although this requires verification by immunohistochemical approaches.

The putative role of the endocannabinoids in modulating activity of neurons in the thalamus in neuropathic rats was also studied. Local thalamic administration of the CB_2_ receptor antagonist SR144528 alone did not significantly alter spontaneous or evoked responses of thalamic neurons in neuropathic rats. By contrast, SR144528 alone significantly increased burst firing of VPL neurons in SNL rats. These data suggest that CB_2_ receptors play a complex role in the tonic control of activity of thalamic neurons. The significance of the lack of effect of CB_2_ receptor antagonism on spontaneous activity compared to burst activity is poorly understood, but it suggests that CB_2_ receptors play a role in tonic inhibition of neuronal activity in the VPL. It has been proposed that burst activity transmits more information than single spikes, and there is a greater probability of a burst than of single spikes generating a single postsynaptic spike (Csicsvari *et al*., 1998). It is also hypothesized that burst firing and single spikes convey different information (Cooper, 2002), such that certain burst parameters convey selective information between neurons (Krahe & Gabbiani, 2004).

Although not the focus of the present study, endocannabinoids may also modulate levels of activity of thalamic neurons via activation of CB_1_ receptors. Indeed, CB_1_ receptors are up-regulated in the thalamus of nerve-injured rats (Siegling *et al*., 2001) and therefore provide an additional target for the endocannabinoids. Levels of AEA, 2AG, PEA and OEA in the thalamus of neuropathic rats were not different from levels in sham-operated rats at 14 days post-surgery, suggesting there are no major changes in endocannabinoid turnover in neuropathic rats at this level. It is important to note, however, that levels of endocannabinoids were measured in the whole thalamus and this may have concealed subtle changes in the levels of endocannabinoids in the VPL of neuropathic rats compared to sham-operated rats. Peripheral nerve damage has been shown to increase levels of AEA, but not 2AG, in the dorsal raphe at 7 days following neuropathic surgery compared to sham-operated controls; however, later timepoints were not studied (Palazzo *et al*., 2006). Similarly, levels of AEA were increased, compared to sham controls, in the spinal cord, periaqueductal grey matter and rostral ventromedial medulla at 7 days following peripheral nerve injury (Petrosino *et al*., 2007). Thus it is clear that there are changes in levels of AEA in the spinal cord and certain brain regions involved in descending inhibitory control pathways at early timepoints following nerve injury, but our data provide no evidence for this at later timepoints in the thalamus, a region involved in ascending pain pathways.

In conclusion, we have demonstrated a functional inhibitory effect of supraspinal administration of the CB_2_ receptor agonist JWH-133, an effect which was blocked by a selective CB_2_ receptor antagonist, in a model of neuropathic pain. These data complement our earlier report of a novel functional role of the CB_2_ receptor in the spinal cord of neuropathic rats compared to sham-operated rats (Sagar *et al*., 2005), and the report that CB_2_ receptor mRNA is present in the spinal cord of neuropathic, but not sham-operated, rats (Zhang *et al*., 2003). Our data suggest that the inhibitory effects of CB_2_ receptor agonists, following nerve injury, are mediated by thalamic as well as spinal (Zhang *et al*., 2003; Sagar *et al*., 2005; Beltramo *et al*., 2006) and peripheral (Elmes *et al*., 2004; Walczak *et al*., 2005; Wotherspoon *et al*., 2005; Walczak *et al*., 2006) sites of action. One of the perceived benefits of targeting the CB_2_ receptor for novel analgesics was the purported absence of receptor in the brain and therefore the lack of the psychoactive side-effects (Malan *et al*., 2003) which limit the clinical usefulness of CB_1_ receptor agonists. The role of CB_2_ receptors in the brain of naïve rodents is unclear and requires further investigation.

## Abbreviations

2AG: 2-arachidonyl glycerol
AEA: anandamide
CB_1_: cannabinoid 1 receptor
CB_2_: cannabinoid 2 receptor
OEA: oleoyl ethanolamide
PEA: palmitoyl ethanolamide
PSTH: peri-stimulus time histogram
SNL: spinal nerve-ligated
VPL: ventral posterior nucleus of the thalamus.
